# iPSC-Derived Microglia for Modeling Human-Specific DAMP and PAMP Responses in the Context of Alzheimer’s Disease

**DOI:** 10.3390/ijms21249668

**Published:** 2020-12-18

**Authors:** Ivanna Ihnatovych, Barbara Birkaya, Emily Notari, Kinga Szigeti

**Affiliations:** Department of Neurology, State University of New York at Buffalo, 875 Ellicott St., Buffalo, NY 14203, USA; ivannai@buffalo.edu (I.I.); barbarabirkaya@gmail.com (B.B.); emilynot@buffalo.edu (E.N.)

**Keywords:** CHRFAM7A, neuroinflammation, Alzheimer’s disease, NF-κB activation, cholinergic anti-inflammatory response, DAMPs, PAMP

## Abstract

Neuroinflammation in Alzheimer’s disease (AD) has been the focus for identifying targetable pathways for drug development. The role of amyloid beta (Aβ), a prototype of damage-associated molecular patterns (DAMPs), has been implicated in triggering an inflammatory response. As alpha7 nicotinic acetylcholine receptor (α7 nAChR) binds Aβ with high affinity, α7 nAChR may play a role in Aβ-induced neuroinflammation. The conundrum of how α7 nAChR as the mediator of the cholinergic anti-inflammatory response may trigger an inflammatory response has not been resolved. *CHRFAM7A*, the uniquely human fusion gene between *ULK4* and *CHRNA7*, is a negative regulator of α7 nAChR ionotropic function. To provide the human context, isogenic induced pluripotent stem cell (iPSC) lines were developed from *CHRFAM7A* null and carrier individuals by genome-editing the null line using TALENs to knock-in *CHRFAM7A*. In iPSC-derived microglia-like cells, CHRFAM7A mitigated Aβ uptake through the α7 nAChR. Despite the lower Aβ uptake, the presence of CHRFAM7A was associated with an innate immune response that was characterized by NF-κB activation and NF-κB target transcription (*TNFA*, *IL6*, and *IL1B*). LPS, a prototype PAMP, induced a heightened immune response in CHRFAM7A carriers. CHRFAM7A modified the dynamics of NF-κB translocation by prolonging its nuclear presence. CHRFAM7A modified the α7 nAChR metabotropic function, resulting in a human-specific innate immune response. This iPSC model provided an opportunity to elucidate the mechanism and establish high throughput screens.

## 1. Introduction

Neuroinflammation has emerged as a targetable mechanism in Alzheimer’s disease (AD) over the last ten years, which has been driven by GWAS (Genome Wide Association Studies) signals in several genes (*CR1, CLU, TREM2, HLA-DRB5/DRB1, INPP5D*, and *MEF2C*) that are implicated in inflammation [1]. Amyloid beta (Aβ), a prototype of damage-associated molecular patterns (DAMPs), has been linked to neuroinflammation. While the role of Aβ in neuroinflammation is complex, the interaction between Aβ and α7 nAChR has produced a conundrum [2,3,4,5]. α7 nAChR is expressed throughout the brain in most cell types, including neurons, microglia, and astrocytes [6]. The α7 nAChR localization pattern correlates with areas in the human brain that are affected in early AD pathology [7]. α7 nAChR binds Aβ with high affinity and Aβ is considered an agonist [8]. On the other hand, α7 nAChR is the mediator of the cholinergic anti-inflammatory response in macrophages [3] and has an anti-inflammatory effect on microglia [4,5]. Of note, the complex interaction between the nervous and immune systems is required in in vivo physiological experimentation using animal models; thus, the cholinergic anti-inflammatory system emerged from animal observations. While α7 nAChR has been an active drug target in AD for over a decade, the vast cumulative preclinical and clinical data revealed one of the most consistent translational gaps between animal models and human trials [9].

The role of human factors in biology and their imposed barriers to modeling human disease in animals was recently demonstrated in AD, specifically for neuroinflammation [10]. Distinctly different transcriptional signatures were detected in human AD and the 5XFAD mouse model of Aβ accumulation [10], with the most marked differences found in microglia. In mice, Aβ triggers a disease-associated microglia (DAM) gene expression signature that consists of the upregulation of *Apoe*, *Trem2*, *Lpl*, *Lilrb4a*, *Cst7*, *Csf1*, *H2-d1*, *Cd74*, and cathepsin genes [10]. In contrast, in human AD, microglia demonstrate the upregulation of glial cell migration, IL-6, and NF-κB signaling, comprising three of the top five upregulated pathways [10]. This observed discordance in mechanisms between humans and mice underscores the importance of understanding human-specific mechanisms.

Duplicate genes that have emerged since the human–chimpanzee divergence are of interest due to their potential to contribute to human-specific traits [11,12]. *SRGAP2C* and *ARHGAP11B* are human-specific duplications with phenotypic readouts that affect the human brain. *SRGAP2C* increases the dendritic spine density [13], while *ARHGAP11B* has been implicated in the expansion of the number of cortical neurons [14]. Both of these human-specific genes carry only a segment of the ancestral gene due to incomplete segmental duplication, suggesting that the truncation of genes is a mechanism that contributes to neofunctionalization [15]. A large-scale functional annotation of human-specific genes revealed their role in immune adaptation, development of the human brain, and adaptation involving metabolic processes [16].

*CHRFAM7A* is a fusion gene between *CHRNA7* and *FAM7A/ULK4* and is only present in humans. The consensus in the literature suggests that *CHRFAM7A* is a negative regulator of the α7 nAChR [17]. Origins of the CHRFAM7A duplication involve complex and sequential chromosomal rearrangements, likely under selective pressure [17]. The chromosomal structure results in ongoing instability, resulting in neurological phenotypes [12,17]. Modeling the function of *CHRFAM7A* requires the human context, which has been unexplored. While the role of *CHRFAM7A* in inflammation and neurodegeneration has been reported in transgenic mouse models, interpretation needs to exercise caution [18,19]. The incorporation of CHRFAM7A as one or two subunits of the α7 nAChR pentamer results in a decreased channel opening probability [20,21] and a prolonged channel inactive state, which facilitates the metabotropic pathway activation [22]. As α7 nAChR is the mediator of the cholinergic anti-inflammatory response through mononuclear cells of the innate immune system [23], it is plausible that CHRFAM7A modifies the innate immune response through an α7 nAChR mechanism in microglia, which are the resident macrophages of the brain. We hypothesized that the incorporation of CHRFAM7A into α7 nAChR results in a hypomorphic ionotropic receptor, resulting in a metabotropic shift that releases the anti-inflammatory tone on NF-κB, and as such, promotes a proinflammatory response. We tested the hypothesis in microglia-like (MGL) cells that were differentiated from the human parent and isogenic induced pluripotent stem cells (iPSCs) to provide the human context [20,21]. To recapitulate the complexity of the human brain, these genome-edited iPSC lines can serve as a genotype-specific 3D model of human tissue, including cerebral organoids and 3D bioprinting [24,25]. Alzheimer’s disease has been challenging to model in animals in general and has resulted in disappointing translational outcomes regarding drug discovery [26]. The complexity, the temporal and spatial resolution, and cell-type diversity of the human brain are oversimplified in 2D cell culture systems [24]. The combination of the human environment using hiPSCs (human iPSCs) and the emergence of 3D modeling technology may facilitate exponential progress in drug discovery with direct human translation [27].

## 2. Results

### 2.1. CHRNA7 and CHRFAM7A Expression in Functional Microglia Like Cells

**Figure 1:**
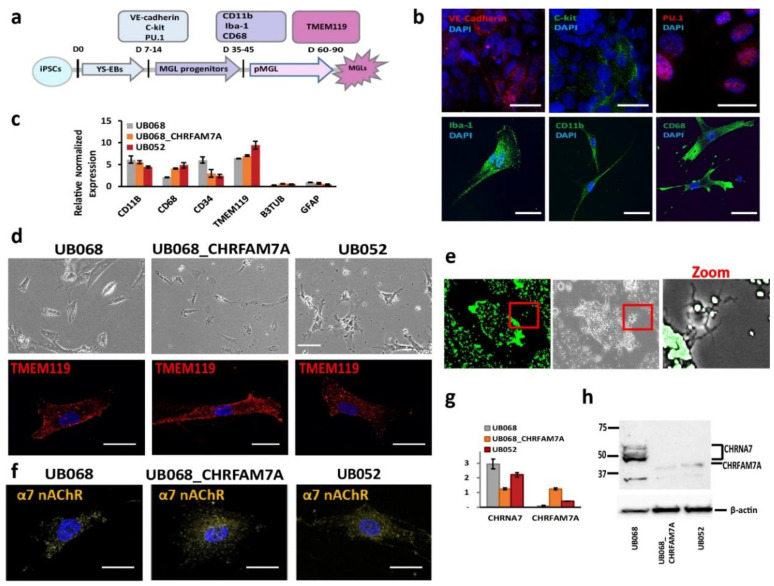
Characterization of microglia-like (MGL) cells derived from CHRFAM7A carrier and non-carrier induced pluripotent stem cell (iPSC) lines: (**a**) Schematic timeline depicts the stages of the MGL differentiation from the iPSC lines and the stage-specific markers used for the characterization of MGL cells. (**b**) Representative confocal images of the yolk sac embryoid bodies (YS-EBs; D 14) plated on PDL-laminin (upper panel) and the MGL progenitors (D 30; lower panel). Scale bar, 20 µm. (**c**) RT-qPCR demonstrates gene expression profiles of the MGL cells (D 60) differentiated from the CHRFAM7A carrier and non-carrier iPSC lines (*n* = 5; three independent cultures). (**d**) Live (phase-contrast microscopy, upper panel; scale bar, 100 µm) and stained MGL cells with microglia marker TMEM119 (lower panel; scale bar, 20 µm). (**e**) Representative images depict the intracellular accumulation of phagocytosed fluorescent beads and the cleared area around the MGL cells (t = 24 h; scale bar, 100 µm). Higher magnification of the area in the red frame is shown on the right. (**f**) Confocal images demonstrate the surface expression of α7 nAChR in MGL cells derived from the iPSC lines. Scale bar, 20 µm. (**g**) RT-qPCR indicates that the presence of CHRFAM7A decreased the expression levels of CHRNA7 in CHRFAM7A carrier lines (*n* = 5; three independent cultures). (**h**) Immunoblot (Millipore-Sigma, Cat# AV35409) demonstrated the expression of CHRNA7 and CHRFAM7A in MGL cells derived from the three iPSC lines. Note that CHRFAM7A was detected only in the UB068_CHRFAM7A and UB052 lines (*n* = 5; three independent cultures).

UB068 (CHRFAM7A null), UB068_CHRFAM7A (isogenic CHRFAM7A carrier), and UB052 (nascent CHRFAM7A carrier) were differentiated into MGL cells through yolk sac embryoid bodies (YS-EBs) in a chemically defined medium that was supplemented with colony-stimulating factor (CSF1) and interleukin IL-34 (Figure 1a). The YS-EBs demonstrated VE-cadherin and c-Kit expression (Figure 1b, upper row), signifying early YS myelogenesis [28], and transcription factor PU.1, which drives microglial differentiation and maintenance [29,30]. Pooled and plated MGL progenitors were positive for surface markers CD11b, CD68, and intracellular calcium-binding protein Iba-1 (Figure 1b, lower row). The MGL cells differentiated from the three iPSC lines expressed microglia-specific markers (Figure 1c) and demonstrated a characteristic morphology (Figure 1d, upper row) and a positive stain with microglia-specific transmembrane protein TMEM119 (Figure 1d, lower row). The functional activity of the MGL cells was demonstrated via phagocytic activity using fluorescent latex beads (Figure 1e).

Immunostaining (ICC) with an antibody generated the epitope in the loop of the α7 nAChR sequence (Millipore-Sigma, Burlington, MA, USA), and thus, detecting both CHRNA7 and CHRFAM7A proteins demonstrated the membrane expression of α7 nAChR (Figure 1c) in microglia derived from CHRFAM7A carrier and non-carrier iPSC lines (Figure 1f). RT-qPCR with CHRNA7- and CHRFAM7A-specific primers targeting the unique breakpoint sequence demonstrated the expression of CHRNA7 on day 40 of the microglial differentiation in all three cell lines. CHRFAM7A expression was detected in abundance in the carrier lines (UB068_CHRFAM7A and UB052 MGL cells) on day 40 of the microglial differentiation (Figure 1g). Western blot analysis confirmed the CHRFAM7A expression in the carrier lines using an antibody against the breakpoint sequence in CHRFAM7A (Millipore-Sigma, Cat# AV35409) (Figure 1h).

### 2.2. CHRFAM7A Mitigated the α7-nAChR-Dependent Aβ_1–42_ Uptake

**Figure 2:**
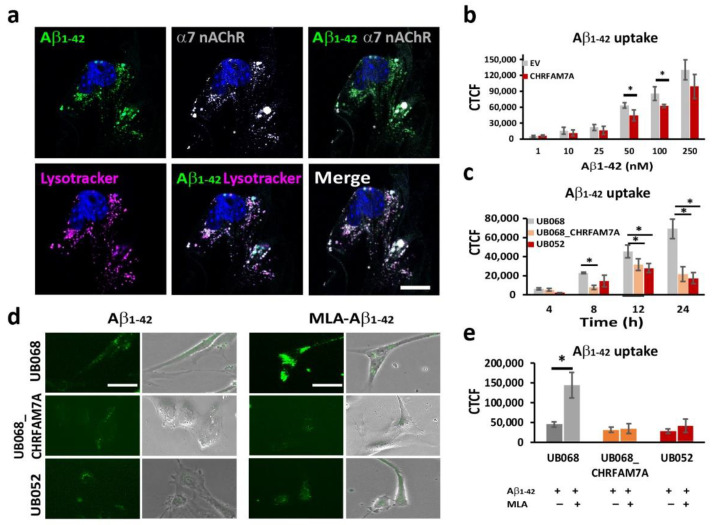
CHRFAM7A mitigated the α7-nAChR-dependent Aβ_1–42_ uptake: (**a**) Confocal microscopy images demonstrated the localization of Aβ_1–42_ (green), LysoTracker Deep Red (magenta), and α7 nAChR (grey) in MGL cells derived from the UB068 line. Nuclei were stained with DAPI (blue). The uptake of Aβ_1–42_ co-occurs with LysoTracker and α7 nAChR. Scale bar, 10 µm. Cells were incubated with Aβ_1–42_ (100 nM) for 24 h, then washed and incubated with LysoTracker for 5 h (*n* = 5; three independent cultures). (**b**) Transfection of the MGL cells derived from UB068 line (0 copy) with CHRFAM7A mitigated the Aβ_1–42_ uptake in a dose-dependent manner. Quantification is presented as the corrected total cell fluorescence (CTCF) using ImageJ. Data are presented as mean ± SD. * *p* < 0.05 (*t*-test)—comparison between the Aβ_1–42_ uptake in CHRFAM7A and EV-transfected cells (50 cells measured from 5 fields; 4 independent cultures; quantification was performed by two blinded raters; *n* = 5; 3 independent cultures). (**c**) Time course of the Aβ_1–42_ uptake (100 nM) by MGL cells that were derived from iPSC lines (UB068, UB068_CHRFAM7A, and UB052). Data are presented as mean ± SD. * *p* < 0.05 (*t*-test)—comparison between UB068 and carrier UB068_CHRFAM7A and UB052 lines at each given time point. (**d**) Representative images of MGL cells derived from the three lines and treated with MLA (10 µM), followed by treatment with Aβ_1–42_ (100 nM) for 18 h (*n* = 5; three independent cultures). (**e**) Pretreatment with MLA (10 µM) increased the Aβ_1–42_ (100 nM) uptake in UB068, but not in CHRFAM7A carrier cell lines. Data are presented as mean ± SD. * *p* < 0.05 (*t*-test)—difference between the Aβ_1–42_ uptake in MGL cells that were pre-treated with MLA in comparison to no pretreatment (*n* = 5; three independent cultures).

Fluorescent Aβ_1–42_ internalized by the microglia partially co-occurred with the α7AChR (Manders’ coefficient: 0.575) and the acidic compartment delineated by Lysotracker (Manders’ coefficient: 0.783) (Figure 2a). The dose-dependent Aβ_1–42_ uptake in MGL cells was characterized using transient transfection of UB068 with pcDNA3.1-CHRFAM7A-mCherry cDNA (CHRFAM7A) compared to pcDNA3.3-mCherry empty vector (EV). We detected a mitigated dose–response curve for Aβ_1–42_ concentrations between 1 nM and 250 nM in the presence of CHRFAM7A, reaching statistical significance at 50 and 100 nM (Figure 2b). The time course of the Aβ_1–42_ uptake (100 nM) in MGL cells derived from the UB068, UB068_CHRFAM7A, and UB052 lines demonstrated a mitigated uptake, as well in the carrier lines (UB068_CHRFAM7A and UB052), compared to the non-carrier line (UB068) (Figure 2c). Pretreatment of MGL cells with the α7AChR-selective antagonist MLA resulted in an increase in Aβ_1–42_ uptake in UB068 (Figure 2d,e), suggesting that MLA blocked the anti-inflammatory effect that induces MGL activation and Aβ_1–42_ uptake, independent of α7AChR. MLA had a minimal effect on the Aβ_1–42_ uptake in the carrier lines (Figure 2d,e).

### 2.3. Immune Surveillance to DAMP: CHRFAM7A Facilitated Microglia Activation

**Figure 3:**
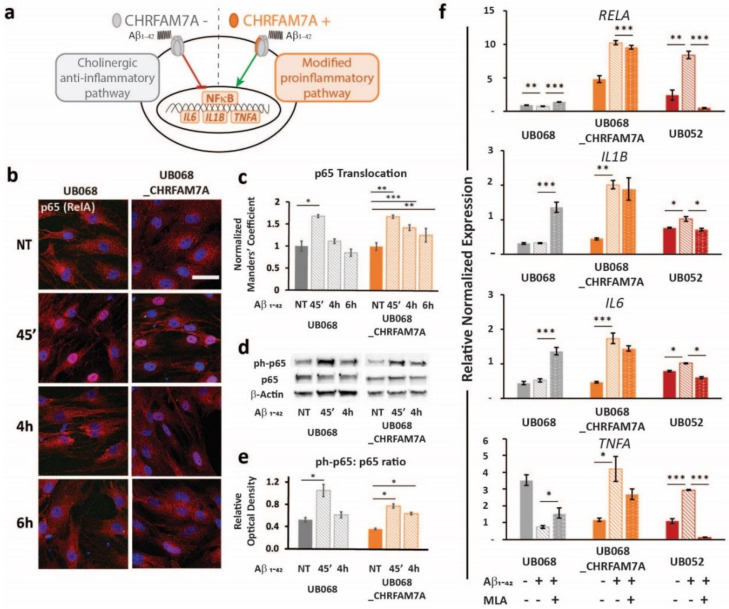
Immune surveillance of DAMP: CHRFAM7A facilitated microglia activation: (**a**) Schematic of the proposed model indicating that CHRFAM7A harboring hypomorphic α7 nAChR released NF-κB inhibition, and as a result, triggered an inflammatory response. (**b**) Representative confocal images demonstrated p65 (RelA) staining during the time course of the Aβ_1–42_ (1 μM) treatment in MGL cells derived from the UB068 (left panel) and UB068_CHRFAM7A (right panel) lines (*n* = 5). Scale bar, 20 µM. (**c**) NF-κB translocation dynamics upon the Aβ_1–42_ treatment up to 6 h using the operator-independent Manders’ coefficient (5 images, at least 100 cells/image) in the UB068 and UB068_CHRFAM7A lines. Data are presented as mean ± SD. * *p* < 0.05, ** *p* < 0.01, *** *p* < 0.001 (*t*-test) - difference in the Aβ_1–42_-induced inflammatory response in comparison to the non-treated controls. (**d**) Representative immunoblots showed an increase in the p65 (RelA) phosphorylation in response to the Aβ_1–42_ treatment in MGL cells derived from the carrier and non-carrier lines. (**e**) Densitometric analysis of the phospho-p65 (Cell Signaling Technology, Cat# 3033) and p65 expression levels in untreated and Aβ_1–42_-treated (1 μM; 45′, 4 h) MGL cells. Data are presented as mean ± SD. * *p* < 0.05 (*t*-test)—difference in Aβ_1–42_-induced inflammatory response in comparison to the non-treated controls (*n* = 5). (**f**) Gene expression of the NF-κB targets *RELA* (top row), *IL1B* (second row), *IL6* (third row), and *TNFA* (bottom row) in response to the Aβ_1–42_ treatment in the UB068, UB068_CHRFAM7A, and UB052 lines. Pretreatment with an α7nAChR-specific antagonist MLA (10 µM) decreased *RELA*, *IL1B*, *IL6,* and *TNFA* expression levels in the CHRFAM7A carrier lines, but increased them in UB068. Data are presented as mean ± SE. * *p* < 0.05, ** *p* < 0.01, *** *p* < 0.001 (*t*-test)—difference in the Aβ_1–42_-induced gene expression levels compared to non-treated controls in the presence and absence of MLA in the three cell lines (*n* = 5).

A schematic of the CHRFAM7A effect on α7AChR-mediated NF-κB inhibition is depicted in Figure 3a. In both lines, the treatment of MGL cells with Aβ_1–42_ (1 μM, 45′, 4 h, 6 h) led to a significant p65 (RelA) translocation to the nucleus, indicating NF-κB activation (Figure 3b, c). Furthermore, the time course experiment suggested that the presence of CHRFAM7A resulted in a prolonged p65 nuclear presence. Manders’ coefficient (M1) (red over blue) in random visual fields captured using confocal microscopy served as an operator-independent quantification of the phospho-p65 co-occurrence with nuclear stain DAPI (blue). Quantification of the complete visual field as a whole incorporated the characteristic cell-to-cell variability of NF-κB translocation into the readout. Manders’ coefficient was validated using the nuclear-to-cytoplasmic ratio of single cells (*n* = 48). Saturation of the Manders’ coefficient occurred at a nuclear-to-cytoplasmic ratio of 0.81. The linear correlation extended to a Manders’ coefficient of 0.81, demonstrating a correlation coefficient of 0.80 between the two methods (Supplementary Figure S3).

The activation of NF-κB was confirmed using an immunoblot. While the level of phospho-p65 was significantly increased by the treatment within a short time (1 μM Aβ_1–42_ 45′) in both lines, it remained elevated after 4 h of Aβ_1–42_ treatment only in the carrier line (Figure 3d,e). Treatment with Aβ_1–42_ triggered NF-κB target transcription (*RELA*, *IL1B*, *IL6*, and *TNFA*) in the carrier lines (UB068_CHRFAM7A and UB052) and decreased RELA and TNFA transcription in the non-carrier line (Figure 3f). The α7AChR antagonist MLA modified the NF-κB target activation in a CHRFAM7A-dependent manner. In the absence of CHRFAM7A (UB068), MLA released the anti-inflammatory tone, inducing an increase in NF-κB target transcription. In the CHRFAM7A carriers, the MLA effect was less robust and worked in the opposite direction, reducing NF-κB target transcription. These data suggest that pure α7AChR mediated the cholinergic anti-inflammatory tone and it was an active process. On the other hand, in the presence of CHRFAM7A, the anti-inflammatory tone was already released by the hypomorphic receptor, thus MLA only had a partial effect.

### 2.4. CHRFAM7A Heightened the Immune Responsiveness to PAMP

**Figure 4:**
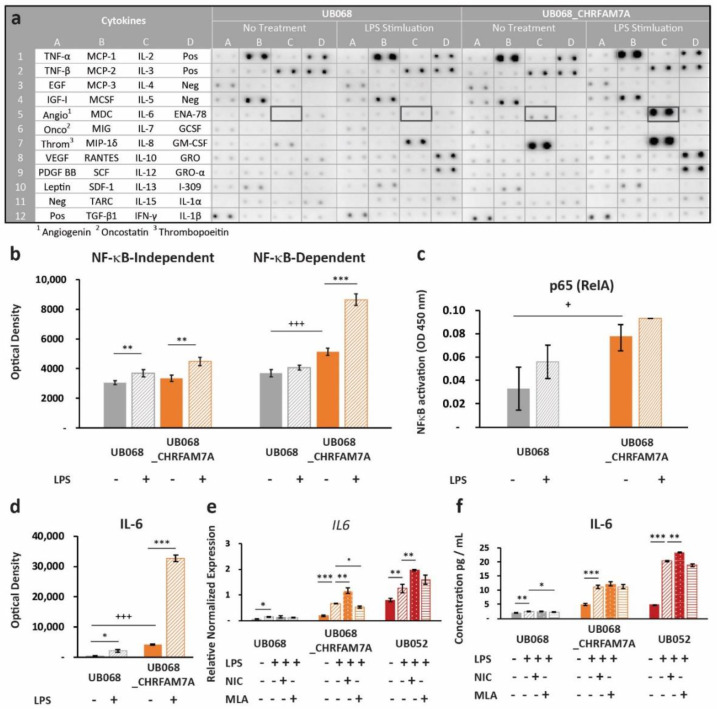
Immune surveillance of PAMPs: (**a**) Human cytokine profiler assay of MGL-conditioned media (24 h) from UB068 and UB068_CHRFAM7A lines at baseline (left) and after 24 h of LPS stimulation (right). Note the robust increase in the IL-6 expression in response to the LPS (1 μg/mL) treatment in UB068_CHRFAM7A. (**b**) Densitometric analysis of NF-κB-independent (left) and NF-κB -dependent (right) cytokines in the LPS treated and untreated MGL cells. Note the difference in the baseline expression level of the NF-κB-dependent cytokines between the two lines. The LPS’s effect on the NF-κB-independent and NF-κB-dependent cytokines is depicted in the non-carrier (UB068) and carrier (UB068_CHRFAM7A) lines. NF-κB-independent cytokines demonstrated similar responses in both lines, while a marked NF-κB-dependent gain of function occurred in the carrier (UB068_CHRFAM7A line). Data are presented as mean ± SD. *** *p* < 0.001—difference in the baseline between the UB068 and UB068_CHRFAM7A lines; ** *p* < 0.01, *** *p* < 0.001 (*t*-test)—difference in the cytokine expression between the LPS-treated and LPS-untreated MGL cells. (**c**) Quantitative analysis of the NF-κB binding in nuclear extracts of the MGL cells derived from the UB068 and UB068_CHRFAM7A lines treated with LPS (1 μg/mL, 3 h). Data are presented as mean ± SD. * *p* < 0.05 (*t*-test). (**d**) Densitometric analysis of IL-6 in media of MGL cells treated with LPS (1 μg/mL, 24 h). Data are presented as mean ± SD. *** *p* < 0.001—difference in the IL-6 baseline between the UB068 and UB068_CHRFAM7A lines; * *p* < 0.05, *** *p* < 0.001 (*t*-test)—difference in the IL-6 expression between the treated and untreated MGL cells. RT-qPCR (**e**) and ELISA (**f**) in the non-carrier (UB068) and carrier (UB068_CHRFAM7A and UB052) lines with pharmacological modulation. Data are presented as mean ± SD. * *p* < 0.05, ** *p* < 0.01, *** *p* < 0.001—difference in the LPS-induced inflammatory response compared to non-treated controls, with and without pharmacological modulation.

Next, we explored whether the proinflammatory switch mechanism applies to other molecular patterns that do not directly bind the α7 nAChR. We selected LPS as a prototype PAMP that interacts with the TLR4 receptor, and at the same time, converges on NF-κB-mediated immune activation. Upon LPS stimulation, we again detected a differential response in the CHRFAM7A carrier (UB068_CHRFAM7A and UB052) and non-carrier (UB068) lines. Human Cytokine Antibody Array (Abcam, Cambridge, U.K.) as a screen for cytokine activation in the non-carrier (UB068) and isogenic carrier line (UB068_CHRFAM7A) (Figure 4a) revealed a higher expression level of NF-κB-dependent cytokines in the isogenic carrier (UB068_CHRFAM7A) line at baseline (Figure 4b). The non-carrier (UB068) line demonstrated NF-κB-dependent and NF-κB-independent immune responses to LPS. The isogenic carrier line (UB068_CHRFAM7A) demonstrated a shift toward an NF-κB-dependent cytokine response after the LPS treatment (Figure 4b). An NF-κB DNA binding assay correlated with both the baseline and LPS-induced NF-κB-dependent cytokine response (Figure 4c). Multiple-testing-corrected differences were detected for IL-8, IL-6, MCP-1 (CCL2), GCSF, MDC (CCL22), GRO-α (CXCL1), ENA-78 (CXCL5), TNF-β, TNF-α, and IL-10 at baseline (Supplementary Figure S1). The cytokine response to LPS included GRO-α (CXCL1), IL-8, and MCP-1 (CCL2) in the non-carrier (UB068) (Supplementary Figure S1). In the isogenic carrier line (UB068_CHRFAM7A), IL-6, GRO-α (CXCL1), TNF-β, IL-8, IL-10, and ENA-78 (CXCL5) showed a significant response to LPS (Supplementary Figure S1). IL-6, IL-8, and MCP-1 (CCL2) emerged as drivers of the NF-κB-mediated effect at baseline and upon LPS stimulation after multiple testing corrections (Supplementary Figure S1, Figure 4d). IL-6 transcriptional (qPCR) (Figure 4e) and cytokine responses (ELISA) (Figure 4f) were validated in the non-carrier (UB068), engineered isogenic (UB068_CHRFAM7A), and nascent carrier (UB052) lines. In the carrier line, nicotine, an α7 nAChR agonist, increased the LPS response by facilitating the proinflammatory effect of CHRFAM7A-modified α7 nAChR (Figure 4e,f). MLA, an α7 nAChR antagonist, had a minimal effect on the inflammatory response as the active process inhibition was already released by the hypomorphic receptor. In contrast, in the non-carrier line (UB068), nicotine facilitated the anti-inflammatory effect and MLA released it (Figure 4e,f). The IL-1β response was below the detection threshold in the cytokine screen but was detected with RT-qPCR and ELISA (Supplementary Figure S2).

### 2.5. CHRFAM7A Affected the NF-κB Translocation Dynamics, Resulting in Innate Immune Activation

**Figure 5:**
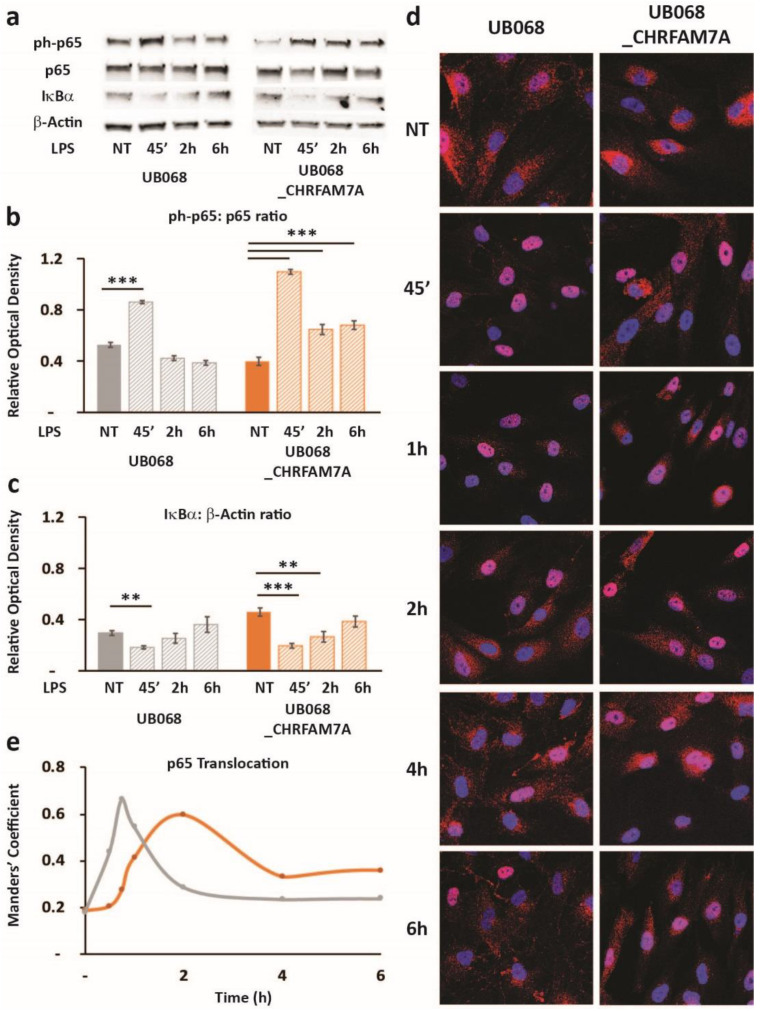
Hypomorphic CHRFAM7A caused NF-κB inhibition, resulting in a proinflammatory switch: (**a**) Immunoblots showed an increase in p65 (RelA) phosphorylation and a decrease in IκBα in response to the LPS treatment (1 μg/mL) in the UB068 and UB068_CHRFAM7A lines. Densitometric analysis of the phospho-p65 (Cell Signaling Technology, Cat# 3033) and p65 (**b**) and IκBα (**c**) expression levels in the untreated and LPS-treated (1 μg/mL; 45′, 2 h, 6 h) MGL cells derived from the UB068 and UB068_CHRFAM7A lines. Data are presented as mean ± SD. ** *p* < 0.01, *** *p* < 0.001 (*t*-test)—difference in the LPS-induced inflammatory response in comparison to non-treated controls (*n* = 5). (**d**) Representative confocal images that demonstrate the p65 (RelA) staining during the time course of the LPS treatment in the MGL cells derived from the UB068 (left panel) and UB068_CHRFAM7A (right panel) lines (*n* = 5). (**e**) NF-κB translocation dynamics upon the LPS treatment up to 6 h using the operator-independent Manders’ coefficient (5 images, at least 100 cells/image) in the UB068 and UB068_CHRFAM7A lines.

Treatment of the MGL cells with LPS led to an NF-κB-induced *RELA* transcription (NF-κB target) in the presence of CHRFAM7A (not shown). The transcriptional response to NF-κB was a highly regulated complex process and was associated with the phospho-p65 translocation dynamic patterns (Figure 5). In the UB068_CHRFAM7A line, the NF-κB target transcription was associated with a distinct time course of the phospho-p65-to-p65 ratio and the IκBa degradation, as detected using an immunoblot (Figure 5a–c). We explored whether the immune activation was associated with the characteristic prolonged NF-κB activation described previously [31]. The MGL cells differentiated from UB068 and UB068_CHRFAM7A were treated with LPS (1 μg/mL) and imaged at 30 min, 45 min, 1 h, 2 h, 4 h, and 6 h. Phospho-p65 was visualized with the anti-p65 (RelA) antibody (red) and counterstained with DAPI to localize the nuclei (Figure 5d). We found that in the presence of CHRFAM7A, the NF-κB translocation curve demonstrated a prolonged nuclear presence, reaching a maximum at 2 h and a sustained NF-κB presence up to 6 h (Figure 5e). In contrast, the NF-κB activation in the non-carrier line (UB068) demonstrated a rapid increase within 30 min with a maximum translocation at 45 min, followed by a rapid exit from the nucleus (Figure 5d,e).

## 3. Discussion

iPSCs as a model system offers a unique opportunity to study cell-type-specific mechanisms with relevance to the human brain. While transgenic animal models revolutionized biology, in AD, their limitations have become apparent, as drugs that showed promise in preclinical models consistently failed in human clinical trials. Uniquely human genes represent one of the mechanisms that account for the human factor and likely create a translational gap. We developed CHRFAM7A carrier and non-carrier human iPSC lines to create a model with this uniquely human context.

While iPSCs can be differentiated to almost any cell type, the differentiation protocols for microglia are still in the dynamic phase to improve the yield, purity, and ease of culturing. For our studies, we used the protocol by Muffat et al. [28] to generate iPSC-derived microglia-like cells. These cells exhibited the transcription factor, gene expression, and protein profiles of microglia. Phagocytosis, a functional characteristic of microglia, was demonstrated by the uptake of fluorescent beads. MGL cells differentiated from the three iPSC cell lines demonstrated similar microglial gene expression signatures and the majority of cells expressed the microglial marker TMEM119. Consistent with literature reports of an increased CHRFAM7A/CHRNA7 ratio in mononuclear cells, including macrophages [32,33], RT-qPCR and immunoblot confirmed CHRFAM7A and CHRNA7 expression in iPSC-derived MGL cells.

Similar to our previous finding in neural progenitor cells (NPCs) [20,21], we found a hypomorphic effect of CHRFAM7A on the Aβ uptake phenotype in MGL cells. In the pure α7 nAChR (absence of CHRFAM7A), Aβ binds to the α7 nAChR with high affinity. As previously demonstrated in NPCs, CHRFAM7A incorporation into the pentamer alters the receptor extracellular domain stoichiometry and results in a hypomorphic receptor with a decreased channel open probability [20,21]. While multiple receptors contribute to Aβ uptake, α7-nAChR-receptor-mediated endocytosis is one of the mechanisms based on the co-occurrence of CHRNA7, Aβ, and lysotracker. Aβ uptake demonstrated a dose–response effect in both the non-carrier and carrier cell lines and mitigated the uptake curve in CHRFAM7A carriers. MLA elicited a paradoxical increase in Aβ uptake in the non-carrier line, suggesting that MLA, an α7 nAChR antagonist, released the anti-inflammatory tone through α7 nAChR and activated the MGL cells. Activated MGL cells appeared to use a non-α7 nAChR mechanism for the Aβ uptake, perhaps phagocytosis or receptor-mediated endocytosis involving Toll-like receptors (TLRs), scavenger receptors (SRs), receptor for advanced glycation end products (RAGE), triggering receptor expressed on myeloid cells 2 (TREM2), or other receptors [34]. In the presence of CHRFAM7A, the MLA effect was marginal, which could be related to the diminished binding of MLA to the hypomorphic receptor harboring CHRFAM7A or other immune effects of the hypomorphic receptor.

Aβ, the prototype DAMP molecule in AD, has been implicated in eliciting a chronic neuroinflammatory response [35]. Parallel to these observations, the association of CHRFAM7A gene expression with the inflammatory response was demonstrated by the PAMP stimulation of macrophages [36]. In our experiments, carrier lines of CHRFAM7A demonstrated an innate immune response upon exposure to Aβ, which was characterized by NF-κB target activation. We found a CHRFAM7A-dependent *RELA*, *IL1B, IL6*, and *TNFA* transcriptional response upon Aβ stimulation. The transcriptional activation was accompanied by NF-κB nuclear translocation. In the absence of CHRFAM7A, the pure α7 nAChR continued to propagate an anti-inflammatory response upon exposure to Aβ, despite higher levels of uptake. In the carrier lines, the inflammatory response was triggered over 50 nM Aβ, corresponding to the physiological concentration upper limit in a human brain [37]. This suggests that CHRFAM7A functions as a tissue surveillance sensor to Aβ. This interpretation is consistent with the microgliosis found in AD brains [10].

These observations suggest that CHRFAM7A shifts the α7 nAChR phenotype from anti-inflammatory to proinflammatory. Existing data on CHRFAM7A suggest that the mechanism is likely an ionotropic-to-metabotropic shift. We and others demonstrated the hypomorphic effect of CHRFAM7A on ionotropic receptor function in transfected oocytes and iPSC-derived neurons, respectively [20,38]. CHRFAM7A decreases the channel open probability and extends the inactivated receptor time. According to the working hypothesis of the α7 nAChR function [22], the prolonged inactive state facilitates the metabotropic activation, which converges on NF-κB. CHRFAM7A transforms the α7 nAChR into a hypomorphic receptor for the ionotropic effect, while at the same time, it activates the metabotropic pathway and releases NF-κB inhibition, resulting in a proinflammatory shift. In the non-carrier line (UB068), which aligns with the animal models, the effect of MLA on the increasing Aβ uptake through immune activation is indicated by the increase in IL-1β, IL-6, and TNF-α. This pharmacological readout is consistent with the paradigm stating that the cholinergic anti-inflammatory tone is the active process, as demonstrated by the release of inhibition with the pretreatment of α-bungarotoxin in other animal models [4,39]. In humans, CHRFAM7A has a similar effect on α7 nAChR, as it releases the active anti-inflammatory tone by shifting from ionotropic to metabotropic action.

This is a common theme in the human-specific fusion gene repertoire, where a human-specific fusion gene functions as a negative regulator of a canonical pathway, resulting in a biological gain of function with a selective advantage. One example is *SRGAP2*, which underwent two human-specific duplications *SRGAP2B* and *SRGAP2C*. The human-specific partial duplications encode a truncated F-BAR domain. SRGAP2C dimerizes with ancestral SRGAP2, inhibiting the block in radial migration. As a result, there is sustained radial migration that is likely contributing to the size evolution of the human neocortex. Another well-characterized uniquely human partial duplication is *ARHGAP11B*. In the transgenic mouse neocortex, *ARHGAP11B* promotes basal progenitor proliferation, enlarges the cortical plate, and induces gyrification. Large-scale functional annotation of human-specific genes revealed their role in immune adaptation, development of the human brain, and adaptation involving metabolic processes [16].

The question arises whether this innate immune switch might be a broader mechanism beyond an Aβ-induced DAMP response in AD. To explore this possibility, we selected an α7-nAChR-independent PAMP, namely, LPS. LPS binds to the TLR4 receptor and activates the innate immune response through NF-κB target activation. In the non-carrier line (UB068), the cytokine screen demonstrated both NF-κB-dependent and NF-κB-independent immune responses to LPS. The isogenic carrier line (UB068_CHRFAM7A) demonstrated a shift toward an NF-κB-dependent cytokine response after the LPS treatment. The NF-κB DNA binding correlated with both the baseline and LPS-induced NF-κB-dependent cytokine response. These data aligned with the hypothesis that hypomorphic α7 nAChR partially reduces NF-κB inhibition, constitutively setting a heightened immune responsiveness state for NF-κB-dependent mechanisms.

IL-6, IL-8, and MCP-1 (CCL2) emerged as drivers of the NF-κB-mediated effect at baseline and upon LPS stimulation. These three cytokines are NF-κB targets and have been implicated in the LPS-induced inflammatory response [40]. The unexpected TNF-α and TNF-β downregulation and IL-10 activation raise the possibility of a complex immune reset and provide future direction to understand these mechanisms. The transcriptional regulation of TNF-α and TNF-β is complex. There is less direct evidence of an NF-κB effect and responses are variable based on the cell type and immune stimulation [41]. IL-10 is considered an anti-inflammatory cytokine by promoting M2 transition [42] and is among the NF-κB-regulated genes [43]. It is expressed in microglia and has been implicated in neuroprotection [44]. Alternatively, these findings could be related to the model and its limitations, which could be clarified by a population-based study using human monocyte/macrophage explants. The IL-6 response was validated with RT-qPCR and ELISA using the pharmacological modulation of α7 nAChR. The agonist (nicotine) and antagonist (MLA) treatment effects on IL-6 transcription, although partial, support the α7-nAChR-mediated proinflammatory switch hypothesis.

As NF-κB is the central transcription factor for mediating the cholinergic anti-inflammatory response, we set out to examine the LPS-induced *RELA* transcription (NF-κB target), NF-κB activation, and translocation dynamics in the absence and presence of CHRFAM7A. We detected a higher *RELA* expression at baseline and in response to LPS in the CHRFAM7A carrier line, similar to that of the NF-κB dependent cytokines. The phospho-p65-to-p65 ratio revealed marked phosphorylation occurring in the CHRFAM7A carrier line when treated with LPS. Furthermore, we detected distinct patterns of NF-κB nuclear translocation dynamics in CHRFAM7A null and carrier MGL cells. CHRFAM7A prolonged the NF-κB nuclear presence compared to the non-carrier line (UB068). The prolonged versus narrow area under the curve dynamics have been associated with distinct transcriptional patterns in single-cell [31] and modeling experiments [45,46], with the prolonged nuclear presence being associated with innate immune activation. The baseline NF-κB nuclear presence and nuclear phospho-p65 levels were similar in CHRFAM7A non-carrier and carrier lines, while the baseline p65 DNA binding and NF-κB transcription were higher in the CHRFAM7A carrier line, suggesting transcriptional priming.

We proposed a model in which hypomorphic-receptor-mediated partial NF-κB release presents a heightened immune responsiveness state, which is then followed by a more robust immune activation in the presence of CHRFAM7A. Elucidating the upstream regulation of NF-κB dynamics requires further studies, where candidates include Ca^2+^ storage regulators, the use of selective Ca^2+^ decoders, Ca^2+^ decoder regulation, and IκK regulation [45,47,48].

Recent work demonstrating the difference in disease-associated microglia (DAM) response to Aβ in humans and mice [10,49] is consistent with our observations. Mice represent the CHRFAM7A non-carrier state, while over 99% of human samples are CHRFAM7A carriers [17]. When comparisons were made between the human and mouse DAM response irrespective of TREM2, the top five upregulated pathways in the human AD brain included chemotaxis, IL-6, and NF-κB, corresponding to the readout from the CHRFAM7A-carrier iPSC model. These findings support the hypothesis that CHRFAM7A infers a human-specific innate immune response that contributes to neuroinflammation in AD. Further understanding of this uniquely human immune mechanism, and its impact on AD, in terms of whether it is protective or destructive would open new areas of treatment strategies with the potential for resolving the translational gap. This iPSC resource is adaptable for high throughput 3D modeling of human tissue by creating cerebral organoids and 3D bioprinting [24,25]. The combination of the human environment using hiPSCs and the emergence of 3D modeling technology may facilitate qualitative progress in drug discovery in general [27]. Furthermore, in the context of a uniquely human gene, functional readout in the human cellular and tissue environment is paramount. Drug development in human 3D tissue equivalents is likely more to be prone to artefacts compared to in vivo animal models; however, with appropriate controls and orthogonal experimental methods, this can be overcome [24]. While the experimental phase is more involved, emerging drug candidates from 3D human screens will likely result in a more direct human translation. Our study has limitations and further larger-scale investigations are needed. The hypothesis needs to be tested in MGL cells derived from a population-representative panel of iPSCs. Postmortem human brain tissue transcriptomic analysis based on CHRFAM7A genotypes may result in additional hypotheses that could be tested in the iPSC model. CHRFAM7A knock-in animal models may or may not replicate these findings, as the human context may be indispensable. However, one of the two published CHRFAM7A KI mouse models has demonstrated effects on monocyte/macrophage differentiation and recovery from a burn injury [18], creating the foundation for cautious optimism.

## 4. Materials and Methods

The materials and reagents are described in detail in the Supplementary Data.

### 4.1. Cell Culture and Microglia Differentiation

UB068 (CHRFAM7A null) and UB052 (nascent CHRFAM7A 1 copy) iPSCs generated from human skin fibroblasts via episomal transformation were described previously [20]. UB068 was genome-edited using TALENs to create isogenic CHRFAM7A-carrier iPSCs (UB068_CHRFAM7A) [21]. Microglia were differentiated according to Muffat et al. [28]. Briefly, iPSC colonies treated with 1 mg/mL collagenase IV and sliced to form uniform clusters were lifted and transferred directly to the microglia-differentiation-defined media that was supplemented with 10 ng/mL CSF1 and 10 ng/mL IL-34 in an ultra-low attachment plate (Corning Inc, Corning, NY). The cells from one confluent six-well plate of iPSCs were pooled into one well of an ultra-low attachment plate. Once the majority of the embryoid bodies (EBs) formed cystic bodies (some phase-bright spheroids were also present), they were triturated and the unsettled supernatant was transferred to a six-well plate. The EBs were triturated five more times and plated into consecutive wells of a Primaria plate. The attached cells were further synchronized using the same microglia differentiation media (MGD) but supplemented with reduced CSF1 (5 ng/mL) and tenfold increased IL-34 (100 ng/mL).

### 4.2. Transfection

MGL cells that were differentiated from the UB068 line were transfected with either pcDNA3.1-CHRFAM7A-mCherry (Addgene plasmid #62635) [50] or with pcDNA3.3-mCherry (Addgene plasmid #26823) [51] constructs using Lipofectamine (Thermo Fisher, Waltham, MA, USA) following the manufacturer’s protocol.

### 4.3. Reverse Transcription Quantitative Polymerase Chain Reaction (RT-qPCR)

Total RNA was isolated using Trizol (Invitrogen). A 500 ng RNA template was used to generate cDNA (ImProm-II reverse transcriptase and oligo (DT) (both Promega, Madison, WI, USA) at 42 °C for one hour. The expressions of CHRNA7 and CHRFAM7A were detected via standard RT-qPCR using specific primers (IDT, Coralville, IA, USA) listed in Table S1 and SYBR green master mix (bimake.com), which were performed on a Bio-Rad CFX Connect cycler (Bio-Rad, Hercules, CA, USA). The relative gene expression was quantified using the ΔΔCT method normalized to GAPDH expression that was assayed with three technical replicates. The results of the triplicate experiments were depicted as mean ± standard error of the mean.

### 4.4. Immunocytochemistry

MGL cells were plated on PDL-Laminin-coated glass coverslips or eight-well glass chambers (Thermo Fisher, Waltham, MA, USA). ICC was performed using standard procedures [20]. Cells were incubated with primary antibodies overnight at 4 °C and with secondary antibodies for 1 h at RT. The primary and secondary antibodies, along with the source and catalog numbers, are listed in Table S2. Confocal images were captured with a Leica TCS SP8 system (63× and/or 20× objective) using LAS X software.

### 4.5. Total Cell Lysate Preparation and Immunoblotting

Total cell lysates from the MGL cells were prepared in a RIPA buffer (Cell Signaling Technologies, Danvers, MA, USA). Western blot analysis was performed according to standard procedures [20,21]. A total of 50 μg of protein was electrophoresed in 4–20% sodium dodecyl sulfate–polyacrylamide gel (Bio-Rad, Hercules, CA, USA), transferred onto polyvinylidene difluoride membrane (Bio-Rad, Hercules, CA, USA), and incubated with primary antibodies overnight at 4 °C (listed in Table S2). ChemiDoc XRS+ Imaging Systems (Bio-Rad, Hercules, CA, USA) detection was followed by densitometry analysis (Bio-Rad Image Lab Software for PC Version 6.1).

### 4.6. Phagocytosis Assay

In vivo phagocytosis in differentiated MGL cells was detected using 1 μm polystyrene yellow-green FluoSpheres (Life Technologies, Carlsbad, CA, USA). A total of 5 μL of FluoSpheres was directly added to MGD media (10^7^ beads/mL/10^5^ MGL cells) and incubated for 24 h. The FluoSpheres engulfment and clearance around the MGL cells were visualized using an EVOS FL microscope (Thermo Fisher, Waltham, MA, USA).

### 4.7. Cytokine Profiler

MGL cells derived from UB068 and UB068_CHRFAM7A lines and grown on six-well plates were treated with 1 μg/mL LPS (or remained untreated) and subjected to a cytokine profiler assay. A total of 1 mL of fresh MGD (with or without LPS) was added to each well (10^5^ cells). Following 24 h of treatment, the media were collected and added to a prepared cytokine antibody panel membrane according to the manufacturer’s protocol (Abcam, Cat#ab133997, Cambridge, MA, USA). The membranes were visualized with ECL ChemiDoc XRS+ Imaging Systems (Bio-Rad) detection was followed by densitometry analysis (Bio-Rad Image Lab Software for PC Version 6.1).

### 4.8. Amyloid Beta Uptake and Cell Counts

Fluor-488- and Fluor-555-labeled Aβ_1–42_ (AnaSpec, Fremont, CA, USA) were reconstituted and the species was confirmed using Beer’s Law (extinction coefficient = absorbance/concentration/path length), as described previously [20,52]. MGL cells plated on PDL-Laminin-coated glass coverslips were treated for 18 h with ascending concentrations (1–250 nM) of HiLyteFluor 488-Aβ_1–42_ for dose–response detection and with 100 nM for the time course. Live images (EVOS FL microscope (Thermo Fisher, Waltham, MA, USA)) captured with a 40× objective were quantified using CTCF in ImageJ, version 1.51 (imagej.nih.gov) [21].

### 4.9. Treatment with LPS and α7 nAChR Agonist/Antagonist

MGL cells differentiated from iPSC lines (UB068, UB068_CHRFAM7A, UB052) were grown in MGD media supplemented with IL-34 (100 ng/mL) and CSF1 (5 ng/mL) on a six-well plate (10^5^ cells/well). Pharmacological modulation consisted of pretreatment with 10 μM nicotine (NIC) or 50 μM methyllycaconitine (MLA) overnight, followed by treatment with 1 μg/mL LPS for 24 h.

### 4.10. ELISA

Following the LPS treatment (with or without pre-treatment with NIC and/or MLA), media were collected. The concentration of IL-6 in the cell culture media was quantified using an IL-6 ELISA kit (R&D Systems, Minneapolis, MN, USA), in keeping with the manufacturers’ protocols.

### 4.11. NF-κB Binding Assay

In MGL cells, the activation of NF-κB in response to the LPS treatment was analyzed via a DNA binding assay using a TransAM^®^ NF-κB p65 Kit (Active Motif, Carlsbad, CA, USA, Cat# 40096) according to the manufacturer’s protocol. Briefly, MGL cells derived from UB068 and UB068_CHRFAM7A and grown in MGD media supplemented with IL-34 (100 ng/mL) and CSF1 (5 ng/mL) on six-well plates (10^5^/well) were treated for 3 h with LPS (1 µg/mL) and nuclear protein extract was isolated. Nuclear extracts were obtained using a nuclear extraction kit (Active Motif, Cat# 40010). A total of 10 µg of nuclear protein extract was applied for the NF-κB binding assay. NF-κB activation was detected by the anti-p65 (RelA) antibody in a colorimetric reaction.

### 4.12. NF-κB Translocation and Quantification

MGL cells differentiated from the UB068 and UB068_CHRFAM7A lines were plated on eight-well glass chambers and treated with Aβ_1–42_ (500 nM) for 4 h or LPS (1 μg/mL) for time points between 30 min and 6 h. The cells were fixed with 4% PFA and ICC was performed with the anti-p65 (RelA) antibody. Confocal images were obtained with 20× and/or 63× objectives (Leica TCS SP8). Four to six random field images for each time point and cell line were quantified using ImageJ (imagej.nih.gov).

To find the Manders’ colocalization coefficient, operator- and fluorescent-intensity-independent high throughput (5 images, >100 cells for each condition) NF-κB translocation quantification was performed using the JACoP plugin in ImageJ. The Manders’ coefficient was calculated (M1) to quantify the amount the red signal (p65) overlapped the blue signal (DAPI). With the cytoplasmic-to-nuclear translocation of NF-κB, the Manders’ coefficient increases. Confocal images were taken with a 20× objective.

The nuclear:cytoplasmic ratio was calculated using the method described in detail in [53] with modifications [54]. Following cell treatment and ICC, the confocal images were taken with a 63× objective. The mean fluorescent signal of phospho-p65 in the nucleus (DAPI positive area) and the cytoplasm (standardized dilated mask of the nucleus) was calculated for randomly selected individual cells (ImageJ). The correlation coefficient for the linear phase between the single-cell Manders’ coefficient and the nuclear:cytoplasmic ratio was calculated for the linear phase.

### 4.13. Statistical Analysis

Experiments were performed in triplicates or quadruplicates. Values are expressed as mean ± SD or ± SE. The unpaired Student’s *t*-test (two-tailed) was performed between comparison groups. Statistical significance was predetermined as *p*-values less than 0.05. For the cytokine screen, Bonferroni multiple testing correction was applied.

## 5. Conclusions

CHRFAM7A provides a uniquely human innate immune response to Aβ by switching the α7 nAChR from an anti-inflammatory to a proinflammatory transducer. This effect is opposite to the α7 nAChR function in preclinical animal models, all of which are non-carriers for CHRFAM7A. α7 nAChR has been implicated in AD for decades; however, this human factor was unknown. Reinterpretation of the wealth of available preclinical and clinical data from this perspective may result in viable drug candidates. Finally, the applicability of the α7-nAChR-dependent proinflammatory switch hypothesis to both α7-nAChR-mediated and α7-nAChR-independent DAMP and PAMP responses suggested a functional role for CHRFAM7A in other areas of innate immunity and may account for the selective pressure leading to the enrichment of CHRFAM7A in the human population.

## Supplementary Materials

The following are available online at https://www.mdpi.com/1422-0067/21/24/9668/s1.

## Abbreviations

α7 nAChR	Alpha7 nicotinic acetylcholine receptor
AD	Alzheimer’s disease
AB	Amyloid beta
DAM	Disease-associated microglia
DAMPs	Damage-associated molecular patterns
PAMPs	Pathogen-associated molecular patterns
MLA	Methyllycaconitine
LPS	Lipopolysaccharides
