# Supplementary material for: iPSC-Derived Microglia for Modeling Human-Specific DAMP and PAMP Responses in the Context of Alzheimer’s Disease
Source: Int J Mol Sci. 2020 Dec 18;21(24):9668. doi: 10.3390/ijms21249668 (PMC7765962; doi:10.3390/ijms21249668)
Supplement: Supplementary file 1 [file ijms-21-09668-s001.zip › ijms-1005874-supplementary/ijms-1005874 supp/ijms-1005874.pptx]

## Slide 1
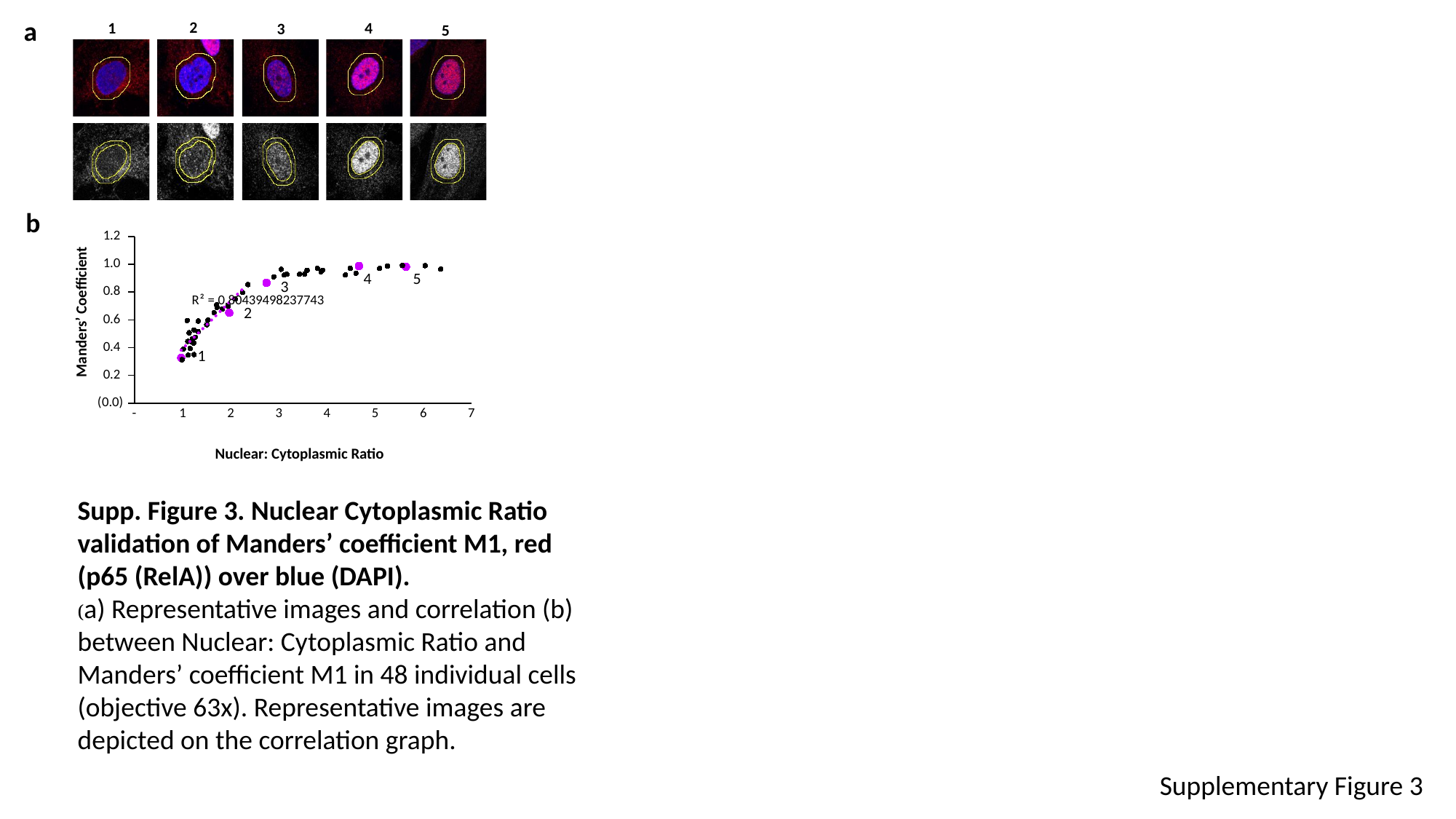

a
2
4
1
3
5
### Chart
| Category | | |
|---|---|---|b
4
5
3
Manders’ Coefficient
2
1
Nuclear: Cytoplasmic Ratio
Supp. Figure 3. Nuclear Cytoplasmic Ratio validation of Manders’ coefficient M1, red (p65 (RelA)) over blue (DAPI).
(a) Representative images and correlation (b) between Nuclear: Cytoplasmic Ratio and Manders’ coefficient M1 in 48 individual cells (objective 63x). Representative images are depicted on the correlation graph.
Supplementary Figure 3
