# Supplementary material for: iPSC-Derived Microglia for Modeling Human-Specific DAMP and PAMP Responses in the Context of Alzheimer’s Disease
Source: Int J Mol Sci. 2020 Dec 18;21(24):9668. doi: 10.3390/ijms21249668 (PMC7765962; doi:10.3390/ijms21249668)
Supplement: Supplementary file 1 [file ijms-21-09668-s001.zip › ijms-1005874-supplementary/ijms-1005874 supp/Supplementary data.pdf]

Supplementary material

Materials and reagents

Cell culture reagents

M-CSF Recombinant Human Protein was purchased from R&D Systems (Minneapolis, MN, USA). Human IL-34 (Carrier free) Recombinant Protein was purchased from Tonbo Biosciences (San Diego, CA). Cell culture media (DMEM/F12 and Neurobasal), supplements (N2, B27), FGF-Basic (AA 1-155) Recombinant Human Protein, chemicals, and all tissue culturing supplies were purchased from Thermo Fisher Scientific.

Cell culture additives

Lipopolysaccharides (LPS), methyllycaconitine (MLA), and Nicotine were bought from MilliporeSigma. Amyloid beta ($A\beta_{1-42}$),

Fluor 488 and Fluor 555 labeled $A\beta_{1-42}$ were purchased from AnaSpec (Fremont, CA).

Plasmids were bought from Addgene (Watertown, MA)

Table S1. Primer Sequences used for RT-qPCR

Gene	Forward Sequence	Reverse Sequence
CD11B	GAAAGGCAAGGAAGCCGGAG	TGGATCTGTCCTTCTCTTAGCCG
CD68	GCTACATGGCGGTGGAGTACAA	ATGATGAGAGGCAGCAAGATGG
CD34	TGCATGTGCAGACTCCTTTC	GAGGACAAGGCTGAGGTCTG
TMEM119	CTTCCTGGATGGGATAGTGGAC	GCACAGACGATGAACATCAGC
GFAP	GTACCAGGACCTGCTCAAT	CAACTATCCTGCTTCTGCTC
IL6	AGTCCTGATCCAGTTCCTGC	CTACATTTGCCGAAGAGCCC
IL1B	GGAGAATGTCCTGAGCACCT	GGAGGTGGAGAGCTTTCAGT
TNFA	GTCAACCTCCTCTCTGCCAT	CCAAAGTAGACCTGCCCAGA
RELA	CTACGACCTGAATGCTGTGC	CTGCCAGAGTTTCGGTTCAC
TUBB3	CAGATGTTCGATGCCAAGAA	GGGATCCACTCCACGAAGTA
GAPDH	GTTCGACAGTCAGCCGCATC	GGAATTTGCCATGGGTGGA

Table S2. Anti-human antibodies used for immunohistochemistry and WB

Antibody	Species	Supplier	Dilution
α-cKit	Rabbit	Cell Signaling Technology, Cat# 3074, RRID:AB_1147633	1:100
α – VE-Cadherin	Mouse	R&D Systems, Cat# MAB9381, RRID:AB_2260374	1:100
α – PU.1	Rabbit	Cell Signaling Technology, Cat# 2266, RRID:AB_10692379	1:100
α – Iba1(AIF-1)	Rabbit	Cell Signaling Technology, Cat#17198T	1:100
α -CD68	Mouse	R&D Systems, Cat# MAB20401, RRID:AB_2074834	1:250
α – CD11b	Rabbit	Novus, Cat# NB110-89474SS, RRID:AB_1216360	1:200
α – TMEM119	Rabbit	Abcam, Cat# ab185333, RRID:AB_2687894	1:100
α – NF-kappaB p65	Rabbit	Cell Signaling Technology, Cat# 8242, RRID:AB_10859369	1:1000 (WB)
α – P-NF-kappaB p65	Rabbit	Cell Signaling Technology, Cat# 3033, RRID:AB_331284	1:400 (ICC) 1:1000 (WB)
α – CHRFAM7A	Rabbit	Millipore-Sigma, Cat# AV35409, RRID:AB_1846696	1:1000
α – α7nAChR	Mouse	Millipore-Sigma, Cat # M220, RRID:AB_260475	1:1000
α – β-actin	Mouse	Millipore-Sigma, Cat# A5316, RRID:AB_476743	1:2000
Alexa Fluor® 594 AffiniPure	Donkey Anti-Rabbit IgG	Jackson ImmunoResearch Labs Cat# 711-585-152, RRID: AB_2340621	1:500
Alexa Fluor® 488 AffiniPure	Donkey Anti-Mouse IgG	Jackson ImmunoResearch Labs Cat# 715-545-151, RRID: AB_2341099	1:500

SUPPLEMENTARY FIGURE LEGENDS

Supp. Figure 1. Quantitative analysis of NFκB dependent and independent cytokine protein expression in MGL cells derived from UB068 (CHRFAM7A null) and UB068_CHRFAM7A lines. Note a significant difference in baseline cytokine expression level (both NFκB dependent and independent) between the CHRFAM7A non-carrier and carrier lines. Treatment with LPS (1µg/ml, 24h) leads to a significant response in cytokine protein expression both in UB068 and UB068_CHRFAM7A lines.

Supp. Figure 2. Quantitative analysis of LPS-dependent IL-1β expression in MGLs differentiated from CHRFAM7A carrier (UB068_CHRFAM7A and UB052) and non-carrier (UB068) lines. (a) RT-qPCR demonstrates a significant increase in *IL1B* expression in response to LPS. Pretreatment with nicotine (NIC) and MLA have opposite effects on LPS induced *IL1B* expression in CHRFAM7A carrier and non-carrier lines. Data are presented as mean ± SD. * - $P < 0.05$, ** - $P < 0.01$, ***- $P < 0.001$ - difference in LPS induced inflammatory response compared to non-treated controls, with and without pharmacological modulation. **(b)** ELISA confirms IL-1β expression in carrier lines correlates with *IL1B.* Data are presented as mean ± SD. * - $P < 0.05$, ** - $P < 0.01$, ***- $P < 0.001$ - difference in LPS induced inflammatory response compared to non-treated controls, with and without pharmacological modulation.

Difference from Baseline:

- IL-8
- IL-6
- MCP-1
- GCSF
- MDC
- GRO-α
- ENA-78 (CXCL5)
- TNF-β
- TNF- α
- IL-10

Response to Treatment (UB068 _CHRFAM7A):

- IL-6
- GRO-α
- TNF-β
- IL-8
- IL-10
- ENA-78 (CXCL5)

Response to Treatment (UB068):

- GRO-α
- IL-8
- MCP-1

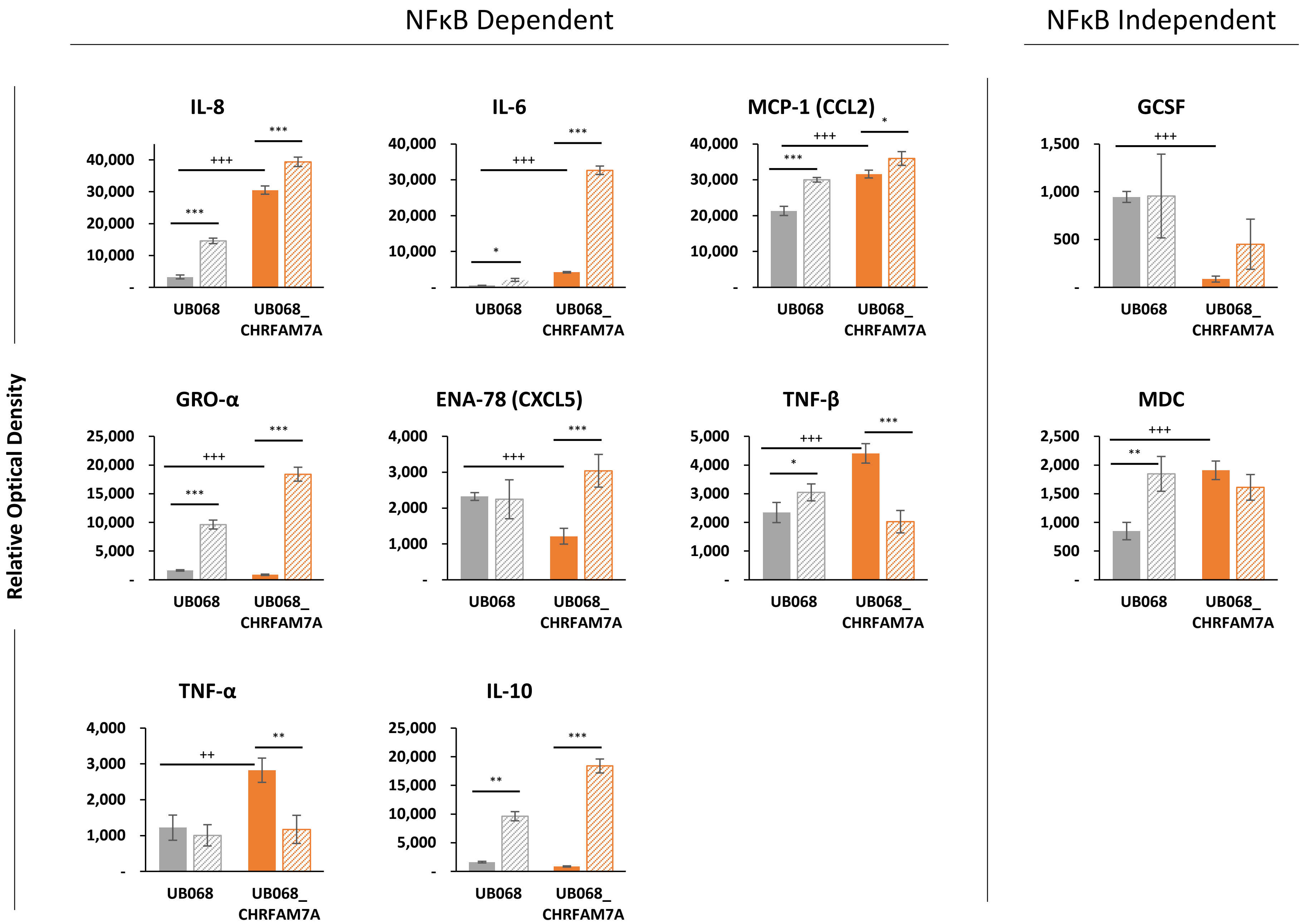

Supplementary Figure 1

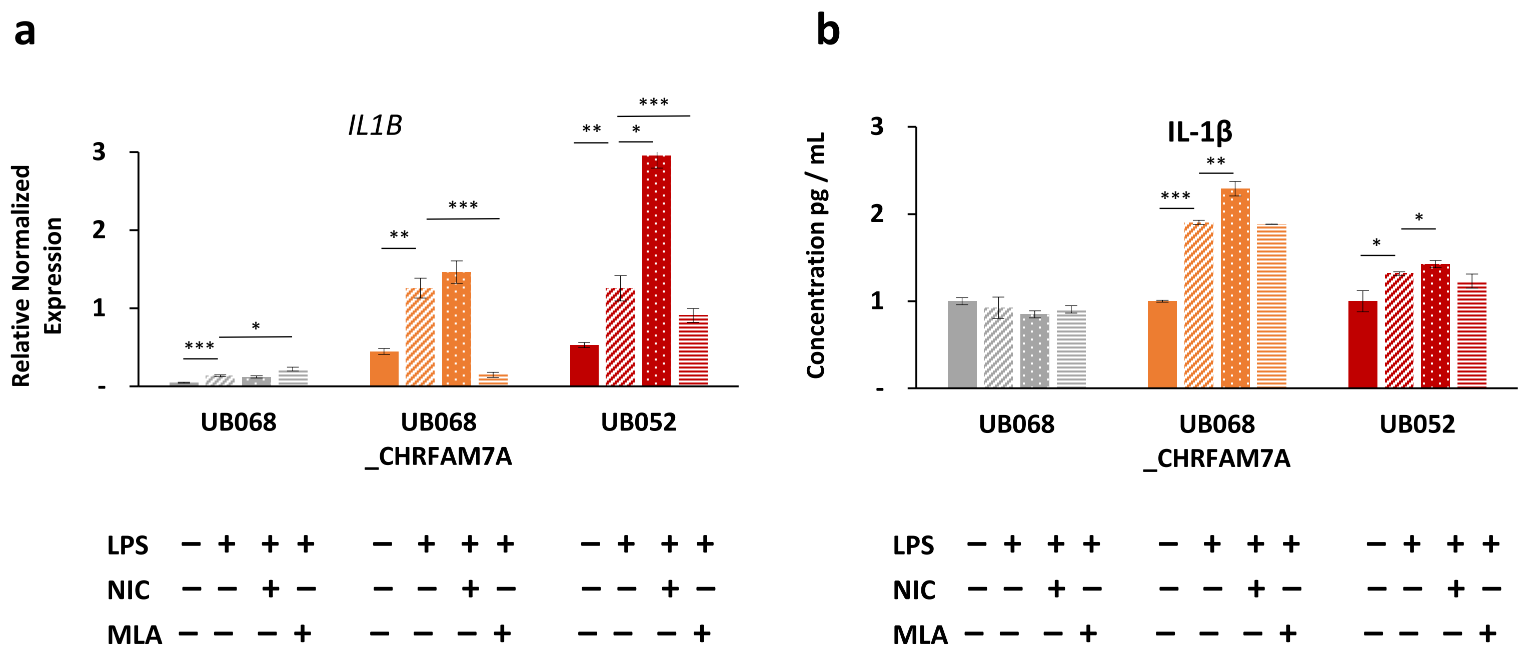

Supplementary Figure 2
